# *Aspergillus* Species and Antifungals Susceptibility in Clinical Setting in the North of Portugal: Cryptic Species and Emerging Azoles Resistance in *A. fumigatus*

**DOI:** 10.3389/fmicb.2018.01656

**Published:** 2018-07-23

**Authors:** Eugénia Pinto, Carolina Monteiro, Marta Maia, Miguel A. Faria, Virgínia Lopes, Catarina Lameiras, Dolores Pinheiro

**Affiliations:** ^1^Laboratory of Microbiology, Biological Sciences Department, Faculty of Pharmacy of University of Porto, Porto, Portugal; ^2^Interdisciplinary Centre of Marine and Environmental Research (CIIMAR/CIMAR), University of Porto, Matosinhos, Portugal; ^3^LAQV-REQUIMTE, Laboratory of Bromatology and Hydrology, Faculty of Pharmacy, University of Porto, Porto, Portugal; ^4^Microbiology Laboratory, Pathology Department, Centro Hospitalar do Porto, Porto, Portugal; ^5^Microbiology Service, Laboratorial Diagnostic Department, Instituto Português Oncologia do Porto Francisco Gentil, EPE (IPOFG-Porto), Porto, Portugal; ^6^Laboratory of Microbiology, Service of Clinical Pathology, Centro Hospitalar S. João EPE, Porto, Portugal

**Keywords:** *Aspergillus* spp., molecular identification, cryptic species, antifungal susceptibility, azole-resistant *A. fumigatus*, *cyp51A* gene

## Abstract

*Aspergillus* spp. are agents of a broad-spectrum of diseases among humans. Their growing resistance to azoles, the cornerstone in the management of human aspergillosis, is a worrisome problem around the world. Considering lack of data from Portugal on this topic, particularly from the northern region, a retrospective surveillance study was planned to assess frequency of cryptic *Aspergillus* species and azoles resistance. A total of 227 clinical isolates, mainly from the respiratory tract (92.1%), collected from three hospitals serving a population of about three million people, were studied for their epidemiology and antifungal susceptibility patterns determined by the E.DEF.9.3 protocol of EUCAST. Employing molecular methods, seven *Aspergillus* complexes were identified; *Aspergillus fumigatus sensu stricto* was the most frequent isolate (86.7%). A 7.5% prevalence of cryptic species was found; *A. welwitschiae* (*A. niger* complex-3.1%) and *A. lentulus* (*A. fumigatus complex*-2.2%) were the most frequent. Amongst cryptic species, it was found a percentage of resistance to voriconazole, posaconazole and isavuconazole of 47.1, 82.4, and 100%, respectively. Five *A. fumigatus sensu stricto* showed pan-azole resistance. Sequencing their *cyp51A* gene revealed the presence of one isolate with TR46/Y121F/T289A mutation and two isolates with TR34/L98H mutation. This study emphasizes the need to identify strains to the species level and to evaluate their antifungal susceptibility in all human originated *Aspergillus* spp. isolates, particularly those from invasive aspergillosis.

## Introduction

*Aspergillus fumigatus* is the leading cause of human aspergillosis, a mold infection that affects immunocompetent and particularly immunocompromised patients. It manifests as a broad-spectrum of diseases including aspergilloma, allergic bronchopulmonary aspergillosis (ABPA), chronic pulmonary aspergillosis (CPA), and invasive aspergillosis (IA), the most critical clinical picture.

In the last two decades, other non-*A. fumigatus* namely *A. flavus, A. terreus*, and *A. niger* and cryptic species have been isolated with increased frequency as causative agents (Lass-Flörl et al., 2005; Krishnan et al., 2009; Alastruey-Izquierdo et al., 2012; Steinbach et al., 2012). Such change in epidemiology has been attributed to the increase in number of the immunocompromised populations, advances in detection, identification of fungal pathogens and the selective pressure caused by the extensive use of broad-spectrum antifungal agents (Richardson and Lass-Flörl, 2008; Alastruey-Izquierdo et al., 2012).

The high diversity of the involved species, their antifungal susceptibility patterns and the severity of the pathology require fast and reliable methods for identification. In the genomic era, molecular techniques became indispensable tools to complement macroscopic and microscopic exam. Methods that use the internal transcribed spacer (ITS), the β-tubulin and the calmodulin regions, are of utmost importance to discern between cryptic species, considered as members of the same complex and, therefore, exhibiting almost indistinguishable morphological properties (Peterson, 2012). Moreover, accurate identification at the species level is crucial, as the emergence of cryptic species is associated with specific patterns of antifungal susceptibility that influence patient management strategies and treatment outcomes (Balajee et al., 2007; Richardson and Lass-Flörl, 2008).

The cornerstone in the management of human aspergillosis is azole treatment. Voriconazole (VCZ) is recommended as the primary agent for IA or CPA, and alternative therapies are isavuconazole (ICZ), liposomal amphotericin B (L-AMB) and anidulafungin (ANI); posaconazole (PCZ) and VCZ are also recommended for prophylaxis. For treatment of ABPA, itraconazole (ITZ) is the first choice and VCZ and PCZ are the second ones (Patterson et al., 2016). Also recently, (Ullmann et al., 2018) published an extensive and detailed article regarding diagnosis and management of aspergillosis, summarizing recommendations of ESCMID, ECMM and ERS. However, since the 90's, reports of azole resistance in *Aspergillus* spp., particularly *A. fumigatus*-complex, became more frequent and now extends to the six continents (Mosquera and Denning, 2002; Howard et al., 2009; Van der Linden et al., 2015; Meis et al., 2016). Several resistance mechanisms have been reported in *A. fumigatus* strains, being mutations in the *cyp51A* gene one of them (Vermeulen et al., 2012). The presence of TR34/L98H mutation has been documented since 2007, and more recently, the emerging TR46/Y121F/T289A mutant has been founded in some countries (Verweij et al., 2007; Vermeulen et al., 2012; Meis et al., 2016). Two possible ways for resistance acquisition to azoles are recognized: “*in vivo*,” as consequence of azole treatment for long periods and associated to a single mutation; “*de novo*,” that seems to occur due to the widespread use of demethylation inhibitors in agriculture and frequently associated to a combination of a tendem repeat (TR) in the promoter region of *cyp51A* and amino acid mutation(s) (Hagiwara et al., 2016; Meis et al., 2016; Garcia-Rubio et al., 2017). Thus, antifungal susceptibility testing of clinical *Aspergillus* isolates is an important source of information in order to avoid therapeutic failures.

In the clinical setting, an important issue is the presence of cryptic species and the emergence of drug resistant *Aspergillus* strains. Thus, the purpose of the present retrospective epidemiological work was to analyse the distribution and characterize the species of *Aspergillus* collected from clinical setting in order to evaluate the presence of cryptic species, their susceptibility patterns to antifungal drugs, and the emergence of resistant strains, which are still unknown in Portugal, particularly in the northern region. In addition, we investigated the underlying resistance molecular mechanisms of *A. fumigatus* strains.

## Materials and methods

### Sample collection

A total of 227 *Aspergillus* clinical strains isolated between January 2010 and March 2016, from biological samples belonging to 207 patients with proven or probable infections or colonization, were studied. They were admitted to the Centro Hospitalar do Porto (HSP1) and Centro Hospitalar São João (HSP2), two tertiary teaching hospitals, and Instituto Português de Oncologia do Porto (HSP3), a hemato-oncological hospital; all of them located within 10 km of distance from each other and serve a population of three million people in north of Portugal.

### Strains identification: morphological and molecular methods

The strains, preserved in 20% Sabouraud broth-glycerol at −80°C, were grown on Sabouraud dextrose agar (SDA) for 3–4 days at 35°C in aerobic conditions. Morphological identification was performed using standard microbiological methods (De Hoog et al., 2001). The macroscopic features and the microscopic morphology were considered. For molecular identification at species level, the International Society for Human and Animal Mycology-sponsored *Aspergillus* Working Group recommendations were followed: amplification of a portion of the β-tubulin gene using primers Bt2a (5′-GGTAACCAAATCGGTGCTGCTTTC-3′) and Bt2b (5′-ACCCTCAGTGTAGTGACCCTTGGC-3′) (Glass and Donaldson, 1995). For some isolates, amplification of a portion of calmodulin gene was performed additionally, using CL1 (5′-GA(GA)T(AT)CAAGGAGGCCTTCTC-3′) and CL2A (5′-TTTTTGCATCATGAGTTGGAC-3′) primers (O'Donnell et al., 2000; Balajee et al., 2007). PCR reactions were performed using a direct DNA amplification kit, *KAPA3G Plant PCR Kit*® (KAPABIOSYSTEMS, Boston, USA). Briefly, one loop of each fungal isolate was collected from 3 to 5 day's cultures in SDA and suspended in 100 μL of ultrapure water. PCR was carried out in a 25 μL volume containing 2x*KAPA3G Plant* PCR Buffer (containing MgCl_2_ and dNTPs); an additional 0.5 mM MgCl_2_; 0.3 μM of each primer, and 0.5 Units of *Taq* DNA polymerase (*KAPA3G Plant* DNA Polymerase). Amplifications were running on a thermal cycler (Bio-Rad, T100^TM^ Thermal Cycler, California, USA) comprising an initial denaturation step at 95°C/15 min followed by 40 cycles of amplification (denaturation at 95°C/20 s, annealing at 58°C/15 s and extension at 72°C/min) and a final extension step at 72°C/1 min. A non-template negative control was included in each amplification reaction. Positive PCR products were purified using the *GRS PCR* & *Gel Band Purification Kit* (Grisp, Porto, Portugal), according to the manufacturer's instructions, and sent to sequencing in both directions with the primers used for amplification. Sequences obtained were manually verified by MEGA7 software package and aligned using the CLUSTALW algorithm. Afterwards, sequences were compared with GenBank and CBS-KNAW Fungal Biodiversity Centre databases. Identifications were based on database rankings and only similarity values ≥99% were considered. The nucleotide sequence representative of each identified species was deposited in the GenBank database (accession numbers from KY696642-47 and KY696649-52).

### Antifungal susceptibility testing

The 227 isolates were tested for *in vitro* antifungal susceptibility using the broth microdilution method as recommended by the protocol E.DEF9.3 of European Committee on Antimicrobial Susceptibility Testing (EUCAST) (EUCAST-E.DEF 9.3)^1^. The reference powders of amphotericin B (AMB, Sigma-Aldrich), L-AMB (Gilead), ANI (Pfizer), caspofungin (CAS, Merck), ITZ (Sigma-Aldrich), VCZ (Pfizer), PCZ (Fluka, Sigma-Aldrich), and ICZ (Basilea Pharmaceutica) were tested with a final concentration of 0.032-16 mg/L. Minimal inhibitory concentrations (MICs) for AMB, L-AMB, and azoles, and minimal effective concentrations (MECs) for ANI and CAS were assessed. Quality control was assured using the strains recommended by EUCAST: *Candida krusei* ATCC6258 and *A. fumigatus* ATCC204305.

Considering the clinical breakpoints (CBPs) and epidemiological cut-offs (ECOFFs), available on EUCAST website (2017), was assessed the presence of resistant (R) and non-wild type (NWT) strains, respectively. For all *Aspergillus* species without CBPs or ECOFFs, were adopted the values established for *A. fumigatus*.

### PCR amplification and sequencing of *A. fumigatus sensu stricto cyp51A* gene

For pan-azoles resistant *A. fumigatus* isolates (HSP3-115, HSP3-32, HSP2-18, HSP3-111, HSP1-6) and for three *A. fumigatus* isolate resistant to ITZ and PCZ (HSP2-67; HSP3-13; HSP3-22) the molecular identification of resistance mechanism was performed in the *cyp51A* gene. Moreover, one randomly *A. fumigatus* isolate without resistance to azoles (HSP2-12) was also analyzed. All isolates were previous identified as *A. fumigatus* by sequencing of part of the β-tubulin gene. The sequencing of *cyp51A* gene and its promotor were obtained, after DNA isolation, using a set of primers, PA-7/PA-5 and P450-A1/P450-A2 (Mellado et al., 2001; Diaz-Guerra et al., 2003). The PCR conditions were performed on the thermal cycler, comprising an initial denaturation step at 95°C/15 min followed by 35 cycles of amplification (denaturation at 95°C/30 s, annealing at 60°C/45 s and extension at 72°C/1 min, for PA-7/PA-5; denaturation at 95°C/30 s, annealing at 58°C/45 s and extension at 72°C/5 min, for P450-A1/P450-A2) and a final extension step at 72°C/1 min. A non-template negative control was included in each amplification reaction. Positive PCR products were purified using the purification kit previously described. The sequencing was done with a set of primers PA-7/PA-5, P450-A2, *cyp51AR3, cyp51AR2* (Mellado et al., 2001; Diaz-Guerra et al., 2003; Prigitano et al., 2014) and a home-designed primer, 5'-GCAGTATGGCGATATCTTCACTT-3'. Sequences obtained were compared with the sequence under accession number AF338659 in GenBank, as wild type reference, in order to find out the mutations. The nucleotide sequence of each *A. fumigatus* isolate studied was deposited in the GenBank database (accession numbers from MH231594-MH231601 and MH040305).

## Results

227 clinical isolates of *Aspergillus* were collected from 207 patients, who included 114 males and 93 females averaging 57.9 years-old (6 months-95 years). The characteristics of each isolate, such as gender, age at the time of sample, diagnostic, collection date, sample source and the molecular identification are presented as Supplementary Material (Table S1). Considering the diagnosis, the most prevalent underlying condition were hemato-oncological (23.2%), oncological (17.4%), lung disease (15.9%), and organs solid transplant (7.2%); most samples (92.1%) were isolated from the respiratory tract (Table S1).

Using morphological methods, six *Aspergillus* complexes were identified, whereas the molecular characterization revealed seven complexes. One isolate was morphologically identified only to the genus level, but molecular methods identified it as *A. sydowii*. Moreover, one misidentification at species complex level was found: morphologically characterized as *A. fumigatus* whilst molecular methods identified as *A. nidulans*.

*A. fumigatus* complex was found to be the most prevalent (86.7%), which is consistent with previous reports (Balajee et al., 2009; Lortholary et al., 2011; Steinbach et al., 2012). Also in agreement with published data (Krishnan et al., 2009; Alastruey-Izquierdo et al., 2012), we observed that the second most prevalent complex was *A. flavus* (6.2%) followed by *A. niger* (3.5%) and *A. terreus* complexes (1.8%).

With the use of β-tubulin sequencing, recognized as a suitable molecular taxonomic target, we were able to identify fungal species within a complex (Balajee et al., 2007; Peterson, 2012). 7.5% of isolates were identified as cryptic species: *A. lentulus, A. thermomutatus, A. felis, A. welwitschiae, A. pseudodeflectus*, and *A. sydowii*. Among them, *A. welwitschiae* (*A. niger* complex-3.1%) and *A. lentulus* (*A. fumigatus* complex-2.2%) were the most frequent. Less frequently *A. thermomutatus* and *A. felis* (*A. fumigatus* complex-0.4% each one); *A. sydowii* (*A. versicolor* complex-0.4%); *A. pseudodeflectus* (*A. ustus* complex-0.9%) were isolated.

MIC values for AMB, L-AMB and the four azoles (ITZ, VCZ, PCZ, and ICZ), and MEC values for ANI and CAS were determined for all isolates. Their range, geometric mean and MICs/MECs distributions are displayed in Table 1. In Table 2 are shown the number and percentage of resistant and NWT *Aspergillus* spp. vs. antifungal drugs, classified using the recently proposed interpretive CBPs and ECOFFs. For ANI and CAS there are not CBPs or ECOFFs established.

**Table 1:** MICs/MECs (mg/L) range, geometric mean (GM) and values distribution of the eight antifungals, according EUCAST protocol.

**Species (n° of isolates)**	**Drug**	**Range**	**GM**	**≤0.032**	**0.064**	**0.125**	**0.25**	**0.5**	**1**	**2**	**4**	**8**	**≥16**
*A. fumigatus* (190)	AMB	0.25–2	1.07				1	29	131	29			
	L-AMB	0.25–2	0.58				28	125	34	3			
	ANI	0.032–0.5	0.19	35	27	54	43	31					
	CAS	0.032–1	0.31	1	24	31	55	77	2				
	ITZ	0.25–16	1.49				5	50	90	37	3		5
	VCZ	0.25–16	0.77				36	96	52	1	3	1	1
	PCZ	0.032–16	0.34	20	21	30	90	21	6		1		1
	ICZ	0.5–16	2.32					1	42	113	31		3
*A. lentulus* (5)	AMB	8–16	11.20									3	2
	L-AMB	16–16	16										5
	ANI	0.125–0.5	0.30			2	1	2					
	CAS	0.5–2	0.90					3	1	1			
	ITZ	16–16	16										5
	VCZ	2–16	6							1	3		1
	PCZ	0.5–16	4.30					1	1	2			1
	ICZ	16–16	16										5
*A. felis* (1)	AMB	2	NA							1			
	L-AMB	4	NA								1		
	ANI	0.5	NA					1					
	CAS	0.5	NA					1					
	ITZ	8	NA									1	
	VCZ	4	NA								1		
	PCZ	0.25	NA				1						
	ICZ	4	NA								1		
*A. thermomutatus* (1)	AMB	1	NA						1				
	L-AMB	0.5	NA					1					
	ANI	0.25	NA				1						
	CAS	0.25	NA				1						
	ITZ	16	NA										1
	VCZ	8	NA									1	
	PCZ	1	NA						1				
	ICZ	4	NA								1		
*A. flavus/oryzae* (14)	AMB	1–4	2.43						4	5	5		
	L-AMB	0.5–16	5.57					2	3	3	1	2	3
	ANI	0.032–16	1.33	1	1	4	6	1					1
	CAS	0.064–8	0.86		4		3	6				1	
	ITZ	0.5–16	2.50					4	3	5	1		1
	VCZ	1–16	2.79						5	7	1		1
	PCZ	0.25–2	0.84				5	1	6	2			
	ICZ	2–16	8							2	3	6	3
*A. welwitschiae* (7)	AMB	0.5–1	0.79					3	4				
	L-AMB	0.125–1	0.41			1	3		2	1			
	ANI	0.064–0.5	0.19		2	2	2	1					
	CAS	0.25–0.5	0.39				3	4					
	ITZ	1–16	4.86						2	2	1	1	1
	VCZ	0.5–1	0.71					4	3				
	PCZ	0.125–2	0.77			1	1	2	2	1			
	ICZ	2–8	4.57							2	3	2	
*A. niger* (1)	AMB	1	NA						1				
	L-AMB	0.25	NA				1						
	ANI	0.5	NA					1					
	CAS	0.5	NA					1					
	ITZ	2	NA							1			
	VCZ	1	NA						1				
	PCZ	0.5	NA					1					
	ICZ	4	NA								1		
*A. terreus* (4)	AMB	4–4	4								4		
	L-AMB	4–4	4								4		
	ANI	0.032–0.064	0.05	1	3								
	CAS	0.25–0.5	0.31				3	1					
	ITZ	0.25–0.5	0.38				2	2					
	VCZ	0.5–1	0.88					1	3				
	PCZ	0.064–0.25	0.17		1	1	2						
	ICZ	2–4	3							2	2		
*A. pseudodeflectus* (2)	AMB	2–2	2							2			
	L-AMB	0.5–1	0.75					1	1				
	ANI	1–2	1.50						1	1			
	CAS	8–8	8									2	
	ITZ	16–16	16										2
	VCZ	16–16	16										2
	PCZ	16–16	16										2
	ICZ	16–16	16										2
*A. nidulans* (1)	AMB	2	NA							1			
	L-AMB	1	NA						1				
	ANI	0.5	NA					1					
	CAS	0.5	NA					1					
	ITZ	1	NA						1				
	VCZ	0.25	NA				1						
	PCZ	0.5	NA					1					
	ICZ	1	NA						1				
*A. sydowii* (1)	AMB	2	NA							1			
	L-AMB	2	NA							1			
	ANI	0.125	NA			1							
	CAS	0.125	NA			1							
	ITZ	16	NA										1
	VCZ	1	NA						1				
	PCZ	1	NA						1				
	ICZ	8	NA									1	

*AMB, amphotericin B; L-AMB, liposomal amphotericin B; ANI, anidulafungin; CAS, caspofungin; ITZ, itraconazole; VCZ, voriconazole; PCZ, posaconazole; ICZ, isavuconazole*.

**Table 2:** Number and percentage of resistant and non-wild type isolates of *Aspergillus* spp. to amphotericin B and four azoles.

	**AMB**		**L-AMB**		**ITZ**		**VCZ**		**PCZ**		**ICZ**	
**Species (n)**	**R (%) CBP>2**	**NWT (%)**	**R (%) CBP>2**	**NWT (%)**	**R (%) CBP>2**	**NWT (%) ECOFF^a^**	**R (%) CBP>2**	**NWT (%) ECOFF^b^**	**R (%) CBP>0.25**	**NWT (%)**	**R (%) CBP>1/0.25^#^**	**NWT(%) ECOFF^c^**
*A. fumigatus* (190)	0	NA	0	NA	8 (4.2)	45 (23.7)	5 (2.6)	6 (3.2)	29 (15.3)	NA	147 (77.4)	34 (17.9)
*A. lentulus* (5)	5 (100)	NA	5 (100)	NA	5 (100)	5 (100)	4 (80)	5 (100)	5 (100)	NA	5 (100)	5 (100)
*A. felis* (1)	0	NA	1 (100)	NA	1 (100)	1 (100)	1 (100)	1 (100)	0	NA	1 (100)	1 (100)
*A. thermomutatus* (1)	0	NA	0	NA	1 (100)	1 (100)	1 (100)	1 (100)	1 (100)	NA	1 (100)	1 (100)
*A. flavus/A. oryzae* (14)	5 (35.7)	NA	6 (42.9)	NA	2 (14.3)	7 (50)	2 (14.3)	2 (14.3)	9 (64.3)	NA	14 (100)	12 (85.7)
*A. welwitschiae* (7)	0	NA	0	NA	3 (42.9)	5 (71.4)	0	0	5 (71.4)	NA	7 (100)	5 (71.4)
*A. niger* (1)	0	NA	0	NA	0	0	0	0	1 (100)	NA	1 (100)	0
*A. terreus* (4)	4 (100)	NA	4 (100)	NA	0	0	0	0	0	NA	4 (100)	4 (100)
*A. pseudodeflectus* (2)	0	NA	0	NA	2 (100)	2 (100)	2 (100)	2 (100)	2 (100)	NA	2 (100)	2 (100)
*A. nidulans* (1)	0	NA	0	NA	0	0	0	0	1 (100)	NA	1^#^ (100)	1 (100)
*A. sydowii* (1)	0	NA	0	NA	1 (100)	1 (100)	0	0	1 (100)	NA	1 (100)	1 (100)

*R, Resistant; NWT, non-wild type; NA, not applicable. AMB, amphotericin B; L-AMB, liposomal amphotericin B; ITZ, itraconazole; VCZ, voriconazole; PCZ, posaconazole; ICZ, isavuconazole. CBP and ECOFF units—mg/L*.

a *ECOFF values for A. niger−4, A. terreus−0.5, A. fumigatus and other species−1*.

b *ECOFF values for A. flavus, A. terreus and A. niger−2, A. fumigatus and other species−1*.

c *ECOFF values for A. niger−4, A. terreus−1, A. nidulans−0.25, A. fumigatus and other species−2*.

Eight azole-resistant *A. fumigatus sensu stricto* isolates were studied. Mutations in the *cyp51A* gene were found in six; the TR34/L98H and TR46/Y121F/T289A mutations, related with resistance to several azoles, were found in three isolates, showing a pan-azole resistance profile. Moreover, other mutations, F46Y/M172V/N248T/D255E/E427K, were found in other three azole-resistant *A. fumigatus* strains and no mutations were observed in two of the isolates (Table 3).

**Table 3:** *Cyp51A* mutations and azoles MICs by EUCAST to *A. fumigatus* isolates.

	***Cyp51A*** **Mutations**									**MIC (mg/L)/EUCAST**			
***A. fumigatus* isolates**	**TR**	**Codon**	**Codon**	**Codon**	**Codon**	**Codon**	**Codon**	**Codon**	**Codon**	**ITZ**	**VCZ**	**PCZ**	**ICZ**
		**46**	**98**	**121**	**172**	**248**	**255**	**289**	**427**				
HSP3-115	–	F46Y	–	–	M172V			–	E427K	4	8	4	4
HSP3-32	TR34	–	L98H	–	–			–	–	≥16	4	0.5	4
HSP2-18	TR34	–	L98H	–	–			–	–	≥16	4	1	≥16
HSP3-111	TR46	–	–	Y121F	–			T289A	–	≥16	≥16	≥16	≥16
HSP1-6	–	–	–	–	–			–	–	≥16	4	1	≥16
HSP3-13	–	F46Y	–	–	M172V	N248T	D255E	–	E427K	4	0.5	1	4
HSP3-22	–	F46Y	–	–	M172V	–	–	–	E427K	4	0.5	1	4
HSP2-67	–	–	–	–	–			–	–	≥16	1	0.5	2
HSP2-12^*^	–	–	–	–	-			–	–	0.5	0.25	0.064	4

*ITZ, itraconazole; VCZ, voriconazole; PCZ, posaconazole; ICZ, isavuconazole*.

* *Not resistant strain. TR-tendem repeat*.

## Discussion

Among filamentous fungi, *Aspergillus* spp. are described as the most important agents of severe opportunistic infections and the genus *Aspergillus* encompasses different clinically relevant complexes: *A. fumigatus* complex, *A. flavus* complex, *A. terreus* complex, *A. ustus* complex and *A. nidulans* complex. In the present work, 227 clinical *Aspergillus* isolates were identified to verify their species distribution and estimate the prevalence of cryptic species in clinical setting.

Like in previous reports, in the present study *A. fumigatus* complex was the most frequently isolated. However, regional differences in the prevalence of these species have been reported: *A. flavus* was described as the second most common *Aspergillus* in medical centers of United States (Balajee et al., 2009), Europe (Alastruey-Izquierdo et al., 2013), Brazil (Negri et al., 2014), and regions with dry weather and/or arid conditions, as the Middle East (Krishnan et al., 2009); *A. terreus* was reported as the second most prevalent in Austria (Lackner et al., 2016); *A. niger* was second in Korea (Heo et al., 2015).

Apart from the identification of the species, these results raise the issue of the cryptic species prevalence in the clinical setting. Although precisely unknown, several reports indicate a frequency of 10–19%, which is slightly higher than the 7.5% of the present research and might be related to the origin of clinical samples, geographical differences or the underlying diseases (Alastruey-Izquierdo et al., 2013; Negri et al., 2014; Sabino et al., 2015).

In clinical settings, an important issue is the susceptibility patterns to antifungal drugs. This applies mainly to cryptic species, frequently reported as resistant, notably to azoles, which emphasize the relevance of their identification and susceptibility testing (Balajee et al., 2007; Alastruey-Izquierdo et al., 2013; Negri et al., 2014; Heo et al., 2015; Lamoth, 2016). However, also important the susceptibility testing for *A. fumigatus sensu stricto*, the most frequently isolated, considering the emergence of resistant strains.

For amphotericin, *A. lenlutus, A. terreus, A. felis* and 5 of 14 *A. flavus/A. oryzae* isolates showed high MIC values, and were categorized as resistant according to the CBPs. Considering the present and previous reported intrinsic resistance or variable susceptibility of these species (Van Der Linden et al., 2011) the therapy with AMB may not be a wise treatment option. AMB and L-AMB showed MIC values ≤ 2 mg/L for all the *A. fumigatus sensu stricto* isolates (190), suggesting overtime stability of AMB susceptibility on this species. The continuous surveillance of *Aspergillus* spp. susceptibility to AMB may properly address this issue.

Regarding ANI and CAS only 1.3 and 3.1% of the isolates showed MEC values higher than 0.5 mg/L, respectively. Although CBPs are not available, the MECs were in general lower for ANI than for CAS and particularly for ANI vs. *A. fumigatus* and their cryptic species, with none value higher than 0.5 mg/L. These observations corroborate ANI utilization on *A. fumigatus* infections, in associations with azoles and/or amphotericin, as recommended by Infectious Diseases Society of America (Patterson et al., 2016).

In general, the azoles' MICs for *A. fumigatus sensu strict*o obtained in this study were higher than previously reported (Perkhofer et al., 2009; Pfaller et al., 2011; Alastruey-Izquierdo et al., 2013; Astvad et al., 2017). However, most of the isolates were susceptible: 95.8% to ITZ, 97.4% to VCZ and 84.7% to PCZ. Although PCZ showed crude lower MICs, these were higher in isolates from the hemato-oncological hospital (21 of the 29 isolates resistant to PCZ, 72.4%), where it is regularly used as prophylactic treatment. Surprisingly, ICZ, a recently introduced antifungal drug, revealed high MIC values. Most of the isolates (59.4%) showed a MIC of 2 mg/L, being categorized as resistant, and 17.9% showed MICs >2 mg/L and were classified as NWT. Etest (data not showed) confirmed these high MIC values and similar results were reported by Arendrup et al. (2017). However, recently, Astvad et al. presented a prevalence of 13.7% of NWT strains to ICZ on a selection of 211 *A. fumigatus* isolates from years 2012-2014 (Astvad et al., 2017). Our prevalence of 17.9%, although higher, may reflect a longer period of evaluation (2010 to March 2016) in a distinct geography. Meanwhile, a recent study indicated that when *A. fumigatus* isolates have MIC = 2 mg/L the treatment of infected patients may still be successful with high doses ICZ (Buil et al., 2018).

Regarding the cryptic species *A. lentulus, A. felis, A. thermomutatus, A. pseudodeflectus* and *A. sydowii*, almost all showed a resistance phenotype to all azoles tested (47.1% to VCZ, 82.4% to PCZ, and 100% to ICZ), which agrees with the literature (Balajee et al., 2007; Richardson and Lass-Flörl, 2008). Exceptions were one isolate of *A. lentulus* categorized as intermediate to VCZ, *A. felis* susceptible to PCZ and *A. sydowii* susceptible to VCZ.

The emergence of *A. fumigatus* with azole-resistant phenotypes has been reported worldwide, resistance being frequently associated with *cyp51A* gene mutations (Verweij et al., 2007; Vermeulen et al., 2012; Meis et al., 2016). Considering mutations' acquisition origin, two routes were established in the last years; environmental, due to the use of azole drugs in agricultural practice; and patient-acquired, due to long periods of azole treatment (Meis et al., 2016; Garcia-Rubio et al., 2017).

Mutations in the *cyp51A* gene were found in six of the eight azole-resistant *A. fumigatus sensu stricto* isolates. The TR46/Y121F/T289A mutant isolate showed very high MIC values to all tested azoles (Monteiro et al., 2018), while the two TR34/L98H mutant isolates showed high MICs to ITZ than other azoles. Other azole-resistant *A. fumigatus* strains evidenced mutations (F46Y/M172V/N248T/D255E/E427K) are likely unrelated to azole resistance and were also reported in susceptible isolates (Howard and Arendrup, 2011). In two other azole-resistant isolates, no *cyp51A* gene mutations were found, suggesting that other mechanisms may be responsible. The source of mutations for these azole-resistant isolates was not established, but according to Meis (Meis et al., 2016) it is probably associated with the environmental route. In Portugal, strains of mutant resistant *A. fumigatus* have never been reported in the environment. However, an increasing prevalence of isolates with high MICs to azoles has suggested a selection of cryptic species with a resistance phenotype after use of antifungal pesticides, such as penconazole (Lago et al., 2014).

Regardless the possible limitations of this study, such as, the relatively low number of studied isolates when compared with reference laboratories (Astvad et al., 2017; Buil et al., 2018), or the lack of clinical details for all patients as prior azoles exposure, immunosuppression status or outcome, the results obtained may contribute to a better understanding of the presence and distribution of cryptic species of *Aspergillus*, their susceptibility patterns to antifungal drugs, the emergence of resistance to antifungals and the spread of mutations associated with azoles resistance.

## Conclusions

The data expressed from this study highlight the presence of *Aspergillus* cryptic species in clinical settings, as well as their resistance to antifungals.

Azole–resistant *A. fumigatus* is a global issue. The emergence and spread of resistance mechanisms, associated with treatment failure in patients with IA, reinforce the need for antifungal susceptibility surveillance as well as to seek out mutations in resistance-linked genes. Moreover, it is important to understand the route of resistance selection in order to implement effective prevention and control measures.

Most *Aspergillus* infections, either with cryptic or resistant strains, affect immunocompromised patients, who are at higher risk for IA. Such important clinical implications emphasize the need to identify the strains to the species level and to perform antifungal susceptibility tests in all *Aspergillus* spp. isolates clinically relevant.

## Author contributions

EP and DP conceived and designed the experiments. EP, DP, and CM wrote the paper. CM, MM, and EP performed antifungal susceptibly tests. CM, MM, and MF performed the molecular identification at species level and sequencing cyp51A gene. DP, CL, and VL collected biological samples and clinical data, isolated and identified the strains by morphological methods. All the authors revised the manuscript and contributed for the final version.

### Conflict of interest statement

The authors declare that the research was conducted in the absence of any commercial or financial relationships that could be construed as a potential conflict of interest.

## Footnotes

^1^EUCAST DEFINITIVE DOCUMENT E.DEF 9.3. Method for the determination of broth dilution minimum inhibitory concentrations of antifungal agents for conidia forming molds. December 2015.
