# Supplementary material for: Aspergillus Species and Antifungals Susceptibility in Clinical Setting in the North of Portugal: Cryptic Species and Emerging Azoles Resistance in A. fumigatus
Source: Front Microbiol. 2018 Jul 23;9:1656. doi: 10.3389/fmicb.2018.01656 (PMC6065200; doi:10.3389/fmicb.2018.01656)
Supplement: Supplementary file 1 [file Data_Sheet_1.PDF]

Table S1 – Gender, age at time of sample, diagnosis, collection date, samples source and molecular identification of the 227 clinical isolates of *Aspergillus* species from three hospitals in the North of Portugal, collected between 2010-2016.

Strain ID	Gender	Age at time of sample	Diagnosis	Collection date	Sample source	Molecular ID
HSP1-C1	M	63	Aspergilloma	01/06/2010	BA	*A. terreus*
HSP1-C2	F	78	Peritonitis	16/10/2011	BAL	*A. welwitschiae*
HSP1-C3	M	88	Chronic obstructive pulmonary disease	22/12/2011	S	*A. niger*
HSP1-C4	F	63	Respiratory infection	22/08/2012	BAL	*A. welwitschiae*
HSP1-C5	F	56	Hepatic transplant	18/09/2012	S	*A. fumigatus*
HSP1-C6	M	45	Sarcoidosis	31/07/2012	S	*A. fumigatus*
HSP1-C7	F	89	Congestive heart failure	15/05/2013	BAL	*A. terreus*
HSP1-C8	M	60	Renal transplant	13/05/2013	S	*A. fumigatus*
HSP1-C9	F	73	Breast cancer	20/05/2013	S	*A. flavus/A. oryzae*
HSP1-C10	M	57	Renal transplant	27/09/2013	S	*A. fumigatus*
HSP1-C11	M	38	Cystic fibrosis	07/11/2013	S	*A. fumigatus*
HSP1-C12	M	63	Chronic obstructive pulmonary disease	28/01/2014	S	*A. flavus/A. oryzae*
HSP1-C13	=C12			28/01/2014		*A. fumigatus*
HSP1-C14	F	80	Respiratory infection	21/03/2014	S	*A. fumigatus*
HSP1-C15	M	45	Silicosis	18/11/2014	BAL	*A. fumigatus*
HSP1-C16	F	48	Hepatic transplant	01/12/2014	BAL	*A. lentulus*
HSP1-C17	F	59	Aspergilloma	03/12/2014	BAL	*A. fumigatus*
HSP1-C18	F	23	Cystic fibrosis	23/12/2014	S	*A. fumigatus*
HSP1-C19	M	64	Chronic obstructive pulmonary disease	16/01/2015	S	*A. fumigatus*
HSP1-C20	M	60	Hepatic transplant	19/01/2015	S	*A. fumigatus*
HSP1-C21	F	9	Cystic fibrosis	26/01/2015	S	*A. fumigatus*
HSP1-C26	=C21			18/05/2015	S	*A. fumigatus*
HSP1-C40	=C21			07/09/2015	S	*A. fumigatus*
HSP1-C22	M	69	Chronic obstructive pulmonary disease	06/02/2015	S	*A. fumigatus*
HSP1-C23	F	63	Hepatic transplant	13/04/2015	S	*A. fumigatus*
HSP1-C24	F	11	Cystic fibrosis	27/04/2015	S	*A. fumigatus*
HSP1-C25	M	16	Cystic fibrosis	04/05/2015	S	*A. fumigatus*
HSP1-C27	M	76	Endophthalmitis	01/06/2015	Vitreous humor	*A. thermomutatus*
HSP1-C28	F	8	Cystic fibrosis	08/06/2015	S	*A. fumigatus*
HSP1-C29	M	72	Renal transplant	23/06/2015	S	*A. fumigatus*
HSP1-C30	F	10	Cystic fibrosis	29/06/2015	S	*A. fumigatus*
HSP1-C31	F	61	Pneumonia	11/07/2015	BAL	*A. fumigatus*
HSP1-C32	M	47	Sepsis	20/07/2015	S	*A. fumigatus*
HSP1-C33	F	57	Sepsis	16/08/2015	S	*A. terreus*
HSP1-C34	M	62	Prolymphocytic leukemia	27/08/2015	S	*A. fumigatus*
HSP1-C35	F	69	Bronchiectasis	09/09/2015	BA	*A. fumigatus*
HSP1-C36	F	81	Pneumonia	27/07/2015	BAL	*A. fumigatus*
HSP1-C37	M	63	Renal transplant	01/08/2015	BAL	*A. fumigatus*
HSP1-C38	M	78	Vascular graft infection	29/09/2015	BA	*A. fumigatus*
HSP1-C39	F	74	Vitreous fibrovascular organization	22/09/2015	Vitreous humor	*A. fumigatus*
HSP1-C41	M	14	Cystic fibrosis	14/09/2015	S	*A. flavus/A. oryzae*
HSP2-C4	M	77	Meningioma	28/11/2013	EA	*A. fumigatus*
HSP2-C1	=C4			22/12/2013	EA	*A. fumigatus*
HSP2-C2	F	69	Lung transplant	30/12/2013	EA	*A. fumigatus*
HSP2-C3	F	77	Spiegelhernia surgery=C38	02/10/2013	EA	*A. fumigatus*
HSP2-C20	=C3			02/10/2013		*A. welwitschiae*
HSP2-C5	M	57	Type 2 diabetes	16/04/2014	BL	*A. fumigatus*
HSP2-C6	M	81	Prostate carcinoma	02/09/2011	S	*A. fumigatus*
HSP2-C7	M	67	Adrenal carcinoma	28/05/2013	S	*A. fumigatus*
HSP2-C8	F	56	Acute respiratory distress syndrome	24/06/2013	EA	*A. fumigatus*
HSP2-C38	M	63	Renal transplant	19/02/2010	EA	*A. fumigatus*
HSP2-C9	=C38			18/02/2012	EA	*A. fumigatus*
HSP2-C10	F	79	Bronchiectasis	06/05/2011	S	*A. fumigatus*
HSP2-C11	F	80	Otitis	02/06/2011	Ear exudate	*A. fumigatus*
HSP2-C12	F	83	ANCA-Positive vasculitis	04/10/2011	BL	*A. fumigatus*
HSP2-C13	M	72	Osteomyelitis	13/05/2011	BL	*A. fumigatus*
HSP2-C14	F	65	Pneumonia	27/03/2013	BAL	*A. fumigatus*
HSP2-C15	F	51	Amyloidosis	16/04/2014	S	*A. fumigatus*

HSP2-C16	F	39	Maxillary sinusitis	07/08/2013	Maxillary sinus drainage	*A. fumigatus*
HSP2-C17	M	78	Chronic myeloid leukemia	11/04/2014	EA	*A. fumigatus*
HSP2-C18	M	71	Colon carcinoma	25/08/2013	EA	*A. fumigatus*
HSP2-C23	=C18			25/08/2013		*A. flavus/A. oryzae*
HSP2-C22	=C18			02/09/2013	EA	*A. flavus/A. oryzae*
HSP2-C19	M	65	Burn wound infection	22/10/2011	EA	*A. lentulus*
HSP2-C26	M	64	Esophageal rupture	05/01/2011	EA	*A. flavus/A. oryzae*
HSP2-C21	=C26			16/01/2011	Mediastinal drainage	*A. welwitschiae*
HSP2-C27	F	29	Acute lymphoblastic leukemia	24/10/2013	EA	*A. flavus/A. oryzae*
HSP2-C25	=C27			14/11/2013	BL	*A. flavus/A. oryzae*
HSP2-C28	F	12	Cystic fibrosis	20/10/2014	S	*A. fumigatus*
HSP2-C29	M	49	Peritonitis	19/11/2014	S	*A. fumigatus*
HSP2-C30	M	43	Cirrhosis	18/12/2014	S	*A. fumigatus*
HSP2-C31	M	19	Cystic fibrosis	20/04/2010	S	*A. fumigatus*
HSP2-C32	F	54	Bronchiectasis	01/07/2010	BAL	*A. fumigatus*
HSP2-C33	M	82	Pancreatitis	23/12/2011	S	*A. fumigatus*
HSP2-C41	=C33			23/12/2011		*A. terreus*
HSP2-C34	M	52	Lung transplant	11/10/2010	S	*A. fumigatus*
HSP2-C35	F	67	Bronchiectasis	21/06/2011	S	*A. fumigatus*
HSP2-C36	F	16	Cystic fibrosis	06/05/2010	S	*A. fumigatus*
HSP2-C37	F	21	Cystic fibrosis	20/10/2014	S	*A. fumigatus*
HSP2-C39	M	38	Burn wound infection	03/09/2010	Burn wound exudate	*A. fumigatus*
HSP2-C40	M	67	Renal transplant	28/10/2013	BL	*A. fumigatus*
HSP2-C42	M	78	Cardiothoracic surgery	16/04/2010	EA	*A. fumigatus*
HSP2-C43	F	37	Cystic fibrosis	07/03/2012	S	*A. fumigatus*
HSP2-C44	M	55	Traumatic brain injury	09/04/2010	S	*A. fumigatus*
HSP2-C45	F	62	Tuberculosis	18/10/2011	EA	*A. fumigatus*
HSP2-C46	F	76	Colon carcinoma	01/10/2014	EA	*A. fumigatus*
HSP2-C47	F	77	Bronchiectasis	13/07/2010	S	*A. fumigatus*
HSP2-C48	F	18	Cystic fibrosis	28/06/2010	S	*A. fumigatus*
HSP2-C49	F	75	Renal transplant	28/12/2014	S	*A. fumigatus*
HSP2-C50	F	47	Bronchiectasis	11/11/2014	Pleural fluid	*A. fumigatus*
HSP2-C51	F	75	Bronchiectasis	27/06/2011	S	*A. fumigatus*
HSP2-C52	M	36	Burn wound infection	08/10/2011	S	*A. fumigatus*
HSP2-C53	F	11	Cystic fibrosis	20/10/2014	S	*A. fumigatus*
HSP2-C54	F	76	Bronchiectasis	26/08/2010	S	*A. fumigatus*
HSP2-C55	F	8	Cystic fibrosis	06/10/2011	S	*A. fumigatus*
HSP2-C56	F	0.5	Cystic fibrosis	13/07/2010	S	*A. fumigatus*
HSP2-C57	F	35	Cystic fibrosis	17/05/2010	S	*A. fumigatus*
HSP2-C58	M	81	Lung adenocarcinoma	05/01/2012	S	*A. fumigatus*
HSP2-C59	M	77	Rheumatoid arthritis	24/08/2010	S	*A. fumigatus*
HSP2-C60	M	61	Thoracic surgery	11/03/2015	Pleural fluid	*A. fumigatus*
HSP2-C64	=C60			30/04/2015	Pleural fluid	*A. fumigatus*
HSP2-C61	M	69	Pneumonia	06/11/2014	BL	*A. welwitschiae*
HSP2-C62	F	37	Bronchiectasis	02/03/2015	S	*A. fumigatus*
HSP2-C63	F	12	Cystic fibrosis	05/03/2015	S	*A. fumigatus*
HSP2-C65	F	63	Sepsis	19/05/2015	S	*A. flavus/A. oryzae*
HSP2-C66	M	47	Lung transplant	20/05/2015	S	*A. fumigatus*
HSP2-C67	M	45	Tuberculosis	27/07/2015	S	*A. fumigatus*
HSP2-C68	F	22	Cystic fibrosis	07/08/2015	S	*A. fumigatus*
HSP2-C69	F	85	ANCA-Positive vasculitis	14/12/2015	S	*A. fumigatus*
HSP2-C70	F	57	Acute myeloid leukemia	19/01/2016	EA	*A. fumigatus*
HSP2-C71	M	60	Acute respiratory distress syndrome	26/01/2016	EA	*A. fumigatus*
HSP2-C72	M	74	Non-Hodgkin lymphoma	18/02/2016	S	*A. flavus/A. oryzae*
HSP2-C73	M	64	Allergic bronchopulmonary aspergillosis	19/02/2016	S	*A. fumigatus*
HSP2-C74	M	54	Renal transplant	19/02/2016	S	*A. flavus/A. oryzae*
HSP2-C75	M	58	Acute respiratory distress syndrome	16/03/2016	EA	*A. welwitschiae*
HSP2-C76	M	60	Pulmonary infiltrates	01/02/2016	Lung biopsy	*A. sydowii*
HSP2-C77	F	81	Peritonitis	02/10/2014	S	*A. lentulus*
HSP3-C1	F	54	Chronic lymphoid leukemia	09/01/2013	S	*A. fumigatus*
HSP3-C2	M	45	Allogeneic bone marrow transplant	06/05/2013	S	*A. fumigatus*
HSP3-C3	M	72	Pneumonia	23/05/2013	EA	*A. fumigatus*
HSP3-C5	F	55	Chronic lymphoid leukemia	04/07/2013	S	*A. fumigatus*
HSP3-C6	F	57	Breast cancer	04/07/2013	EA	*A. fumigatus*

**HSP3-C7**	**M**	**75**	**Pneumonia**	**04/07/2013**	**S**	***A. fumigatus***
**HSP3-C8**	**M**	**76**	**Primary hepatocelular carcinoma**	**06/07/2013**	**S**	***A. fumigatus***
**HSP3-C9**	**M**	**60**	**Myelofibrosis**	**16/07/2013**	**BL**	***A. fumigatus***
**HSP3-C10**	**M**	**59**	**Pneumonia**	**18/07/2013**	**S**	***A. fumigatus***
**HSP3-C11**	**F**	**74**	**Chronic lymphoid leukemia**	**19/07/2013**	**S**	***A. fumigatus***
**HSP3-C12**	**M**	**61**	**Pulmonary nodule**	**19/07/2013**	**BL**	***A. fumigatus***
**HSP3-C13**	**M**	**38**	**Non-Hodgkin lymphoma**	**25/07/2013**	**S**	***A. fumigatus***
**HSP3-C14**	**F**	**70**	**Breast cancer**	**29/07/2013**	**BL**	***A. fumigatus***
**HSP3-C15**	**M**	**82**	**Colon cancer**	**06/08/2013**	**BL**	***A. fumigatus***
**HSP3-C16**	**M**	**75**	**Non-Hodgkin lymphoma**	**30/08/2013**	**BAL**	***A. fumigatus***
**HSP3-C17**	**M**	**65**	**Lung adenocarcinoma**	**04/09/2013**	**S**	***A. fumigatus***
**HSP3-C18**	**M**	**66**	**Lung adenocarcinoma**	**27/09/2013**	**BL**	***A. fumigatus***
**HSP3-C19**	**M**	**75**	**Colon cancer**	**25/10/2013**	**BL**	***A. fumigatus***
**HSP3-C20**	**M**	**57**	**Prostate carcinoma**	**31/10/2013**	**S**	***A. fumigatus***
**HSP3-C21**	**F**	**75**	**Gastric cancer**	**17/11/2013**	**EA**	***A. fumigatus***
**HSP3-C22**	**M**	**68**	**Acute myeloid leukemia**	**26/11/2013**	**BAL**	***A. fumigatus***
**HSP3-C23**	**F**	**78**	**Lung adenocarcinoma**	**14/12/2013**	**S**	***A. fumigatus***
**HSP3-C24**	**M**	**51**	**Invasive aspergillosis**	**31/12/2013**	**BL**	***A. flavus/A. oryzae***
**HSP3-C25**	**F**	**76**	**Pulmonary nodule**	**09/01/2014**	**BL**	***A. fumigatus***
**HSP3-C26**	**M**	**76**	**Chronic lymphoid leukemia**	**09/01/2014**	**BL**	***A. fumigatus***
**HSP3-C27**	**M**	**51**	**Hepatic failure**	**09/01/2014**	**S**	***A. fumigatus***
**HSP3-C28**	**F**	**70**	**Sarcoma**	**28/01/2014**	**BL**	***A. fumigatus***
**HSP3-C29**	**M**	**21**	**Acute myeloid leukemia**	**28/01/2014**	**Fluids**	***A. fumigatus***
**HSP3-C30**	**F**	**71**	**Sarcoma**	**03/03/2014**	**EA**	***A. fumigatus***
**HSP3-C31**	**M**	**62**	**Lung adenocarcinoma**	**08/05/2014**	**BL**	***A. fumigatus***
**HSP3-C32**	**M**	**57**	**Chronic obstructive pulmonary disease**	**30/05/2014**	**S**	***A. fumigatus***
**HSP3-C33**	**M**	**63**	**Post chemotherapy neutropenia**	**04/07/2014**	**S**	***A. fumigatus***
**HSP3-C34**	**F**	**83**	**Thyroid cancer**	**16/07/2014**	**BL**	***A. fumigatus***
**HSP3-C35**	**M**	**83**	**Lung adenocarcinoma**	**16/07/2014**	**S**	***A. fumigatus***
**HSP3-C36**	**M**	**52**	**Pneumonia**	**16/07/2014**	**S**	***A. fumigatus***
**HSP3-C37**	**M**	**84**	**Pneumonia**	**21/07/2014**	**BAL**	***A. fumigatus***
**HSP3-C38**	**M**	**65**	**Cervical cancer**	**22/07/2014**	**S**	***A. fumigatus***
**HSP3-C39**	**M**	**54**	**Tuberculosis**	**22/07/2014**	**S**	***A. fumigatus***
**HSP3-C40**	**M**	**51**	**Graft-versus-host disease**	**24/07/2014**	**S**	***A. fumigatus***
**HSP3-C41**	**M**	**62**	**Non-Hodgkin lymphoma**	**11/08/2014**	**BL**	***A. fumigatus***
**HSP3-C42**	**M**	**60**	**Allogeneic bone marrow transplant**	**20/08/2014**	**S**	***A. fumigatus***
**HSP3-C43**	**M**	**35**	**Acute myeloid leukemia**	**21/09/2014**	**S**	***A. fumigatus***
**HSP3-C44**	**F**	**65**	**Mycetoma**	**29/10/2014**	**BAL**	***A. fumigatus***
**HSP3-C45**	**M**	**41**	**Acute lymphoid leukemia**	**04/11/2014**	**S**	***A. fumigatus***
**HSP3-C46**	**F**	**65**	**Non-Hodgkin lymphoma**	**11/11/2014**	**BL**	***A. fumigatus***
**HSP3-C47**	**F**	**57**	**Non-Hodgkin lymphoma**	**23/11/2014**	**EA**	***A. fumigatus***
**HSP3-C48**	**=C47**			**24/11/2014**	**Biopsy**	***A. fumigatus***
**HSP3-C49**	**M**	**62**	**Pulmonary nodule**	**28/11/2014**	**BL**	***A. fumigatus***
**HSP3-C50**	**F**	**59**	**Pneumonitis**	**01/12/2014**	**BAL**	***A. fumigatus***
**HSP3-C51**	**M**	**64**	**Lung adenocarcinoma**	**11/12/2014**	**BL**	***A. fumigatus***
**HSP3-C52**	**M**	**62**	**Pulmonary nodule**	**16/12/2014**	**S**	***A. fumigatus***
**HSP3-C53**	**F**	**80**	**Atelectasis**	**18/12/2014**	**BL**	***A. fumigatus***
**HSP3-C54**	**F**	**45**	**Breast cancer**	**03/04/2011**	**S**	***A. fumigatus***
**HSP3-C56**	**M**	**52**	**Allogeneic bone marrow transplant**	**23/05/2011**	**EA**	***A. lentulus***
**HSP3-C57**	**M**	**49**	**Lung adenocarcinoma**	**27/05/2011**	**BL**	***A. fumigatus***
**HSP3-C58**	**M**	**74**	**Sepsis**	**02/06/2011**	**S**	***A. fumigatus***
**HSP3-C59**	**F**	**50**	**Pneumonia**	**03/06/2011**	**S**	***A. fumigatus***
**HSP3-C61**	**F**	**41**	**Pulmonary nodule**	**09/10/2011**	**S**	***A. fumigatus***
**HSP3-C62**	**M**	**79**	**Pneumonia**	**23/12/2011**	**S**	***A. flavus/A. oryzae***
**HSP3-C63**	**M**	**69**	**Chronic lymphoid leukemia**	**04/04/2012**	**S**	***A. fumigatus***
**HSP3-C64**	**F**	**73**	**Chronic lymphoid leukemia**	**11/04/2012**	**S**	***A. fumigatus***
**HSP3-C65**	**F**	**79**	**Non-Hodgkin lymphoma**	**17/04/2012**	**S**	***A. fumigatus***
**HSP3-C66**	**M**	**60**	**Acute kidney injury**	**18/04/2012**	**S**	***A. felis***
**HSP3-C67**	**=C66**			**30/04/2012**	**EA**	***A. fumigatus***
**HSP3-C68**	**F**	**17**	**Acute myeloid leukemia**	**02/05/2012**	**S**	***A. fumigatus***
**HSP3-C69**	**M**	**43**	**Septic shock**	**06/05/2012**	**EA**	***A. fumigatus***
**HSP3-C70**	**F**	**71**	**Septic shock**	**06/05/2012**	**Exudate**	***A. fumigatus***
**HSP3-C72**	**M**	**43**	**Gastric cancer**	**19/07/2012**	**S**	***A. fumigatus***
**HSP3-C73**	**F**	**78**	**Lung adenocarcinoma**	**23/07/2012**	**BL**	***A. fumigatus***
**HSP3-C74**	**M**	**75**	**Non-Hodgkin lymphoma**	**26/07/2012**	**S**	***A. fumigatus***

**HSP3-C75**	**M**	**86**	**Pulmonary nodule**	**27/07/2012**	**BL**	***A. fumigatus***
**HSP3-C76**	**M**	**56**	**Renal carcinoma**	**27/07/2012**	**S**	***A. fumigatus***
**HSP3-C77**	**M**	**59**	**Acute kidney injury**	**28/07/2012**	**S**	***A. fumigatus***
**HSP3-C78**	**F**	**81**	**Abdominal surgery**	**24/08/2012**	**S**	***A. fumigatus***
**HSP3-C79**	**M**	**16**	**Septic shock**	**30/08/2012**	**Biopsy**	***A. fumigatus***
**HSP3-C80**	**M**	**46**	**Cervical cancer**	**05/09/2012**	**S**	***A. fumigatus***
**HSP3-C81**	**F**	**84**	**Non-Hodgkin lymphoma**	**10/09/2012**	**Exudate**	***A. fumigatus***
**HSP3-C83**	**M**	**49**	**Allogeneic bone marrow transplant**	**01/10/2012**	**S**	***A. fumigatus***
**HSP3-C84**	**F**	**79**	**Non-Hodgkin lymphoma**	**08/10/2012**	**BL**	***A. fumigatus***
**HSP3-C85**	**M**	**70**	**Colon cancer**	**06/11/2012**	**BL**	***A. fumigatus***
**HSP3-C86**	**M**	**21**	**Non-Hodgkin lymphoma**	**08/01/2015**	**S**	***A. fumigatus***
**HSP3-C87**	**F**	**40**	**Aplastic anemia**	**09/01/2015**	**S**	***A. fumigatus***
**HSP3-C88**	**F**	**83**	**Hemoptysis**	**23/01/2015**	**S**	***A. fumigatus***
**HSP3-C89**	**F**	**51**	**Acute myeloid leukemia**	**16/03/2015**	**EA**	***A. fumigatus***
**HSP3-C90**	**=C89**			**23/03/2015**	**Biopsy**	***A. fumigatus***
**HSP3-C91**	**M**	**70**	**Chronic lymphoid leukemia**	**24/03/2015**	**S**	***A. fumigatus***
**HSP3-C92**	**M**	**40**	**Allogeneic bone marrow transplant**	**30/03/2015**	**S**	***A. fumigatus***
**HSP3-C93**	**F**	**79**	**Sepsis**	**01/04/2015**	**S**	***A. fumigatus***
**HSP3-C94**	**=C93**			**01/04/2015**		***A. fumigatus***
**HSP3-C95**	**M**	**49**	**Chronic lymphoid leukemia**	**06/04/2015**	**BAL**	***A. fumigatus***
**HSP3-C96**	**=C95**			**06/04/2015**	**Biopsy**	***A. fumigatus***
**HSP3-C97**	**F**	**63**	**Cervical cancer**	**07/04/2015**	**S**	***A. fumigatus***
**HSP3-C98**	**M**	**40**	**Allogeneic bone marrow transplant**	**08/04/2015**	**S**	***A. fumigatus***
**HSP3-C99**	**F**	**60**	**Non-Hodgkin lymphoma**	**21/04/2015**	**S**	***A. fumigatus***
**HSP3-C100**	**=C99**			**21/04/2015**		***A. fumigatus***
**HSP3-C101**	**F**	**6**	**Allogeneic bone marrow transplant**	**23/04/2015**	**S**	***A. fumigatus***
**HSP3-C102**	**M**	**37**	**Myelodysplastic syndrome**	**30/04/2015**	**EA**	***A. fumigatus***
**HSP3-C103**	**F**	**66**	**Non-Hodgkin lymphoma**	**20/05/2015**	**S**	***A. fumigatus***
**HSP3-C104**	**M**	**21**	**Acute lymphoid leukemia**	**30/06/2015**	**S**	***A. welwitschiae***
**HSP3-C105**	**M**	**71**	**Non-Hodgkin lymphoma**	**09/07/2015**	**S**	***A. nidulans***
**HSP3-C106**	**F**	**55**	**Pulmonary nodule**	**10/07/2015**	**BAL**	***A. fumigatus***
**HSP3-C107**	**M**	**57**	**Esophagal carcinoma**	**22/07/2015**	**S**	***A. fumigatus***
**HSP3-C108**	**=C107**			**22/07/2015**		***A. flavus/A. oryzae***
**HSP3-C109**	**F**	**55**	**Hodgkin lymphoma**	**24/07/2015**	**S**	***A. fumigatus***
**HSP3-C110**	**M**	**56**	**Cervical cancer**	**27/07/2015**	**S**	***A. lentulus***
**HSP3-C111**	**M**	**52**	**Acute lymphoid leukemia**	**02/09/2015**	**S**	***A. fumigatus***
**HSP3-C112**	**M**	**62**	**Cervical cancer**	**09/09/2015**	**Oral exudate**	***A. fumigatus***
**HSP3-C113**	**F**	**68**	**Myelofibrosis**	**09/11/2015**	**S**	***A. pseudodeflectus***
**HSP3-C114**	**=C113**			**17/11/2015**	**BL**	***A. pseudodeflectus***
**HSP3-C115**	**F**	**95**	**Bladder cancer**	**01/12/2015**	**S**	***A. fumigatus***

HSP1-CHP; HSP2-CHSJ; HSP3-IPO; S-sputum; BA-bronchial aspirate; BL-bronchial lavage; BAL-bronchoalveolar lavage; EA-endotracheal aspirate. Molecular ID by β-tubulin sequencing.
